# Post-COVID-19 Pulmonary Fibrosis: Novel Sequelae of the Current Pandemic

**DOI:** 10.3390/jcm10112452

**Published:** 2021-06-01

**Authors:** Shiva Rattan Ambardar, Stephanie L. Hightower, Nikhil A. Huprikar, Kevin K. Chung, Anju Singhal, Jacob F. Collen

**Affiliations:** 1 Department of Medicine, F. Edward Hebert School of Medicine, Uniformed Services University of the Health Sciences, Bethesda, MD 20814, USA; stephanie.l.hightower.mil@mail.mil (S.L.H.); nikhil.a.huprikar.mil@mail.mil (N.A.H.); kevin.chung@usuhs.edu (K.K.C.); jacob.collen@usuhs.edu (J.F.C.); 2 Department of Pulmonary, Critical Care, and Sleep Medicine, Walter Reed National Military Medical Center, Bethesda, MD 20814, USA; 3 Inova Fairfax Advanced Lung Disease Center, Fairfax, VA 22033, USA; anju.singhal@inova.org

**Keywords:** SARS-CoV-2, coronavirus, pulmonary fibrosis, interstitial lung disease, COVID-19, ARDS, venous thromboembolism, pneumonia, mechanical ventilation

## Abstract

Since the initial identification of the novel coronavirus SARS-CoV-2 in December 2019, the COVID-19 pandemic has become a leading cause of morbidity and mortality worldwide. As effective vaccines and treatments begin to emerge, it will become increasingly important to identify and proactively manage the long-term respiratory complications of severe disease. The patterns of imaging abnormalities coupled with data from prior coronavirus outbreaks suggest that patients with severe COVID-19 pneumonia are likely at an increased risk of progression to interstitial lung disease (ILD) and chronic pulmonary vascular disease. In this paper, we briefly review the definition, classification, and underlying pathophysiology of interstitial lung disease (ILD). We then review the current literature on the proposed mechanisms of lung injury in severe COVID-19 infection, and outline potential viral- and immune-mediated processes implicated in the development of post-COVID-19 pulmonary fibrosis (PCPF). Finally, we address patient-specific and iatrogenic risk factors that could lead to PCPF and discuss strategies for reducing risk of pulmonary complications/sequelae.

## 1. Introduction

The first reports of a novel coronavirus SARS-CoV-2 came from Wuhan, China, in December 2019. As this highly transmissible virus spread rapidly across the globe, it quickly overwhelmed medical and critical care resources, becoming a leading cause of morbidity and mortality worldwide [1]. Declared a global pandemic by the World Health Organization (WHO) on 11 March 2020, coronavirus-19 (COVID-19) has to-date infected over 110 million people worldwide and led to over 2.5 million deaths. 

As the pandemic evolves and effective vaccines and treatments begin to emerge, it is increasingly important to build our understanding of the long-term complications in patients infected with the SARS-CoV-2 virus. Due to the high prevalence of respiratory failure and the need for mechanical ventilation in patients with severe manifestations of the disease, there has been increasing concern about the pulmonary sequelae, most notably pulmonary fibrosis (PF) [2]. Given that survivors of COVID-19 who develop persistent pulmonary disease will require long term specialty care, all clinicians have a vested interest in understanding and mitigating the various risk factors associated with post-COVID-19 pulmonary fibrosis (PCPF). In this paper, we aim to briefly define interstitial lung disease (ILD) and review the underlying pathogenesis leading to the development of pulmonary fibrosis. We then present a summary of the current literature describing both the viral- and immune-mediated mechanisms implicated as primary contributors to lung injury in severe COVID-19 disease. Finally, we address risk factors for progression to PCPF and mitigation strategies.

## 2. Definition and Classification of Interstitial Lung Disease (ILD)

Interstitial lung disease (ILD) is a broad term that includes various diffuse parenchymal lung diseases with a spectrum of clinical, radiologic, and pathologic features. It is often characterized by shared features of inflammation and/or fibrosis [3]. PF, on the other hand, is a pathological outcome of acute and chronic ILDs in which normal regulation of tissue repair is compromised [4,5]. Since PF is a subset of ILDs, PF is always related to ILD. PF is characterized by impaired reconstruction of the damaged alveolar epithelium, persistence of fibroblasts, excessive deposition of extracellular matrix (ECM) components such as collagen, and the destruction of normal lung architecture [4]. The term fibrosis usually implies collagen deposition on histopathology [3,6]. Pathological studies in humans and animal models have also shown the presence of proliferation myofibroblasts and alveolar remodeling [5]. However, histology of post-COVID-19 PF is not well defined.

To clarify some related terms, diffuse alveolar damage (DAD) is not fibrosis in itself, but fibrotic features can be part of DAD. In the fibrotic phase of DAD there is failure of removal of alveolar collagen, which is laid down early in the process of lung injury [7]. Ground glass opacity (GGO) is a radiologic sign which is more likely to represent potentially reversible inflammation, such as from pneumonia, than fibrosis [3,8].

Idiopathic PF (IPF) is a distinct type of fibrotic ILD. Viral infections notably have been associated with developing IPF [9] and are thought to be co-factors in the onset of IPF, although by definition, IPF has no known trigger [10,11]. A meta-analysis showed that the presence of persistent or chronic viral infections significantly increases the risk of developing IPF, but this data did not find a role for coronaviruses [12].

Specific terminology here can create confusion, and clarification of “post-COVID-19 PF” is needed, as it is often used interchangeably to refer to one of the following: post-acute respiratory distress syndrome (ARDS) PF [13], post-inflammatory PF [4], post-viral PF, and post-viral ILD. The etiology, prognosis and progression of post-viral PF syndromes may differ from fibrotic ILDs like IPF [14]. Wallace et al. advocated clarifying nomenclature since fibrosis should refer, by definition, to an irreversible end state; therefore, he argues “fibrosis” should not apply to the abnormalities seen in cases of post-viral PF since these changes could reverse over time [15]. Other authors argue that “fibrosis” can be considered a potentially reversible process, and the term “reversible pulmonary fibrosis” has been used in the current literature [16,17]. In this review, we will use the term “PCPF” to encompass a non-idiopathic form of PF associated with COVID-19 infection, which is heterogeneous in many aspects and can present anytime from initial hospitalization to long term follow-up. However, it is as yet early in the COVID-19 pandemic and there is still much uncertainty about many aspects of this condition.

## 3. Post-COVID Pulmonary Fibrosis

A diagnosis of PCPF should be based on clinical, radiologic, and pathologic information. Lab tests, pulmonary function tests (PFTs), and/or high resolution CT (HRCT) in the setting of a patient with previous or suspected COVID-19 infection may provide evidence to support a diagnosis of PCPF. In light of current circumstances (shortages of personal protective equipment (PPE), medical providers, and procedural space), it is difficult to justify pursuing high-risk, aerosolizing procedures such as bronchoscopy or surgical lung biopsy for diagnosis, especially since these would not change management in the acute setting [18,19]. To date, there are no reliable data on the frequency and severity of PF associated with COVID-19.

Several recent COVID-19 studies have described patients with residual radiographic abnormalities consistent with pulmonary fibrosis [20,21] and concomitant findings of fibrotic features on histopathology [1,22]. Among 90 hospitalized COVID-19 patients, the majority had residual mild to substantial pulmonary changes on CT at discharge, a median of 24 days after symptom onset [20]. Some authors have classified PCPF radiologically based on extensive and persistent fibrotic changes, including parenchymal bands, irregular interfaces, reticular opacities, and traction bronchiectasis with or without honeycombing [23]. Other studies indicated that some follow-up CTs showed extensive fibrosis outright [21] Notably, since there is no single test that proves the diagnosis, it is very important that these radiologic changes occurred temporally associated with recent COVID-19 infection.

There is some variation in presentation and severity, but cases generally present with bilateral GGOs [24], later progressing to fibrosis with negative COVID-19 tests. In a systematic review of 131 pulmonary samples from COVID-19 patients, three histologic patterns of lung injury were identified and often found to be overlapping: epithelial, vascular, and fibrotic [1]. In an autopsy study, the fibrosing DAD pattern was seen most commonly and typically showed either alveolar duct fibrosis or diffuse thickening of alveolar walls [25]. 

## 4. Etiology and Pathophysiology

**Figure 1:**
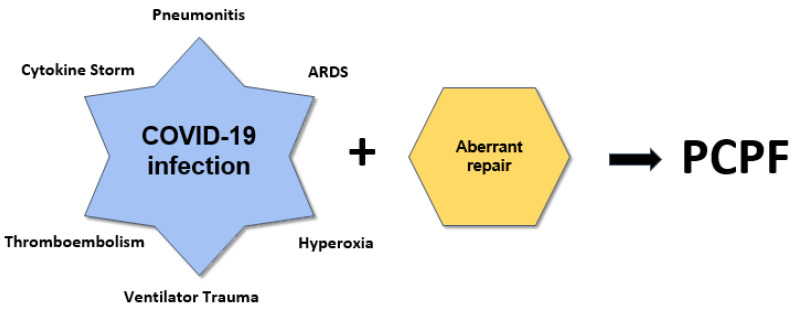
Injury → Inflammatory response → Repair → Fibrosis.

Potential contributing etiologies for PCPF include viral pneumonia and pneumonitis [4,26,27,28,29]; ARDS from COVID-19 pneumonia and COVID-19 related sepsis [4,27,28,30,31]; trauma (prolonged mechanical ventilation (MV)) [13,26,31,32,33]; thromboembolism [4,28]; hyperoxia [4,28,34,35]; and dysregulations in the immune response [4,26,27,28,29,30,31] (Figure 1). These factors may overlap, and notably, trauma from MV is not necessary for PCPF to occur [29]. There has been some discussion on P-SILI (patient-self induced lung injury), a form of lung injury that is thought to occur early in ARDS, in which strong spontaneous breathing effort may contribute to lung damage, and there has been debate on if this should affect timing of intubation [36,37].

### 4.1. Viral Pneumonia and Pneumonitis

Prior to COVID-19, there have been three recent global viral pneumonia outbreaks: SARS coronavirus (SARS-CoV) in 2002, Influenza A H1N1 (2009), and most recently Middle Eastern respiratory syndrome coronavirus (MERS-CoV) in 2012. From 2002–2004, SARS-CoV led to >8000 admissions (20% developed ARDS) with a case fatality rate exceeding 9% [38,39]. ICU mortality for SARS-CoV was reportedly between 35–43%. Among those requiring MV, mortality was reported to be between 52–64% [38,39,40,41,42,43,44,45]. The 2009 Influenza A H1N1 pandemic led to ICU admission in 9–31% of adults and mortality of 14–27% among the critically ill with rates as high as 42% for patients requiring MV [39,46,47,48,49,50,51]. For MERS-CoV, ICU mortality rates have been reported to be between 58–90% and 72–75% among those requiring MV [39,52]. The mortality rates for both MERS-CoV and COVID-19 are particularly alarming. However, global implications for COVID-19 are much direr given the drastic difference in range. The World Health Organization has documented 854 deaths due to MERS-CoV since 2012. COVID-19 by contrast continues to spread with more than 2.5 million deaths globally since January 2019.

Among patients with MERS who were evaluated for residual radiographic findings after recovery, more than 33% had fibrosis. Fibrotic changes were linked to more severe initial radiographic findings on plain chest X-ray (CXR), longer duration of ICU admission (19 days or longer), longer duration of mechanical ventilation, older age, and higher lactate dehydrogenase levels. Patients without fibrosis tended to have more pleural disease or ground glass opacities [39,53]. A paucity of literature has addressed pulmonary sequelae following recovery from COVID-19. Spirometry demonstrating a restrictive pattern weeks after discharge in severe COVID-19 survivors could be due to weakness/deconditioning from prolonged MV or parenchymal disease. One study evaluated COVID-19 survivors 8–12 weeks after illness and only found objective abnormalities (radiography, spirometry, and laboratory) in 35% of the patients. Abnormalities were mostly in those patients with moderate to severe disease who required supplemental oxygen. The majority of patients struggle with residual pulmonary complaints such as dyspnea and cough, often without objective findings on testing [54]. 

### 4.2. Post-ARDS Pulmonary Fibrosis

By contrast, in ARDS survivors there is extensive literature documenting the correlation of physiologic and radiologic data with health-related quality of life (HR-QOL), as well as pulmonary-specific measures. Survivors may have various pulmonary abnormalities including restriction, which may be due to neuro-muscular weakness (NMW) and deconditioning more so than parenchymal injury. Burnham et al. showed the radiographic changes and physiologic measures correlated well with patient’s symptoms and reduced pulmonary function months after diagnosis in a number of acute lung injury (ALI)/ARDS survivors [55]. These patients tended to have low diffusing capacity for carbon monoxide (DLCO) supporting direct pulmonary injury impacting gas exchange [56]. Common variables for fibrotic lung disease following viral respiratory failure are advanced age, prolonged duration of mechanical ventilation, and worsened initial radiographic changes, all of which are consistent with a baseline more severely ill population. The underlying pathophysiology is likely multifactorial, with the largest contributions coming from mechanical ventilation induced trauma to the lungs, as well as aberrant reparative processes. In response to viral mediated lung damage, dysregulation of epidermal growth factor receptor (EGFR) signaling may lead to a prolonged and exaggerated wound healing response, leading to fibrosis [10,57].

### 4.3. Direct Trauma from Mechanical Ventilation

A postulated role of prolonged mechanical ventilation-induced lung injury (VILI) in PF has been outlined by several authors [32]. Although mechanical ventilation (MV) is the most important supportive therapy for ARDS, it can cause or worsen lung injury—which is referred to as VILI [32]. A significant proportion of patients with COVID-19 require MV as a supportive treatment and in one study of 5700 hospitalized COVID-19 patients, 20% required MV [58]. ARDS causing respiratory failure is a frequent cause of morbidity and mortality in COVID-19 patients and often is the reason they need MV [59,60]. The initial inflammatory injury of ARDS to the lung may be augmented by mechanical forces of MV [61]. VILI presents similarly to and is clinically indistinguishable from ALI/ARDS [62]; thus, it is difficult to determine cause and effect and whether the virus, the disease process (ARDS), or the treatment (MV) is the culprit for any ensuing and persistent lung injury [62,63]. 

### 4.4. Thromboembolism

In addition to causing a clinical array of respiratory-related disorders, COVID-19 has also been shown to result in a profoundly prothrombotic state leading to both micro- and macro-thrombotic disease [27]. At present, the specific pathophysiology underlying this hypercoagulable state remains unclear; proposed mechanisms include a combination of hyperinflammatory processes triggering thrombo-inflammation; dysregulation of complement, fibrinolytic and plasminogen systems; and viral-mediated endothelial cell injury [64]. However, this is not specific to COVID-related ARDS; ARDS in general is associated with pulmonary thrombosis and it is not clear that COVID-related ARDS has more or less thrombosis than non-COVID related ARDS. 

Thromboembolism and hypercoagulability may be implicated in pathogenesis of pulmonary fibrosis. Epidemiologic observations have supported this possibility. A large cohort study showed that the incidence rates of ILD were higher in patients with a history of venous thromboembolism or pulmonary embolism than in control patients [6,65]. A possible mechanism would be pulmonary emboli leading to lung injury and damage, triggering or contributing to fibrosis [65]. Grosse et al. evaluated the spectrum of cardiopulmonary histopathology of COVID-19 based on non-minimally invasive autopsies, and their findings revealed different stages of DAD in all fourteen patients assessed, with the presence of thrombotic/thromboembolic vascular occlusions in an overwhelming majority (11/14) [66]. Thus, pulmonary artery thrombi in COVID-19 may be attributable to dysregulation of the inflammatory and reparatory mechanisms as a result of DAD. Prior autopsy series from patients infected with SARS-CoV-1 seem to support this theory as the authors considered fibrin microthrombi in small pulmonary arteries as a common finding of DAD, however, this is a common finding in autopsies of patients with ARDS from other disease states and may simply be a reflection of illness severity. 

### 4.5. Pro-Inflammatory State

Another mechanism more recently hypothesized as a potential contributor to the immune dysregulation and hypercoagulable state found in COVID-19 patients are neutrophil extracellular traps (NETs) [67]. Activated neutrophils have the unique ability to form NETs, which are weblike structures rich in host DNA, modified histone proteins, and granule proteins such as neutrophil elastase (NE) and myeloperoxidase (MPO). Initially discovered for their role in bactericidal activities, NETs are now hypothesized to be involved in a variety of infectious and non-infectious processes that lead to lung damage, thrombosis, and fibrosis. Interestingly, NETs have been found in the airways and pulmonary microcirculation of COVID-19 patients, but were not detected in the lungs of patients who died of other causes [67]. Further investigation is required to more specifically elucidate whether NETs are directly involved in the formation of pulmonary micro-thrombi, but it is possible that under hyper-inflammatory conditions such as those induced by severe COVID-19 infection, NETs could represent a mechanism by which neutrophils contribute to thrombus formation, host-system repair dysregulation, and subsequent pulmonary fibrosis formation. A possible mechanism by which NETs may contribute to PCPF is that in advanced stages, NETs could be replaced by collagen networks [67,68].

Immunological dysregulation, also known as the “cytokine storm”, may be a significant contributor to multiorgan dysfunction. Many cytokines have been reported at elevated levels in COVID-19 cases, including IL1-β, IL-6, IL-7, IL-8, and tumor necrosis factor-α (TNF-α). Elevated proinflammatory cytokines correlate with disease severity [69,70]. The immune induced mechanism of PF is important to address. Immune-related damage contributes to COVID related ARDS. Also, transforming growth factor beta (TGF-β) is a cytokine thought to be a crucial mediator of initiation and progression of fibrosis and remodeling [71]. Its expression is increased in animal models of PF and in human lungs with IPF [5,72]. IL- 6 and IL-16 are other cytokines that may also be implicated in lung or other organs’ fibrosis [73,74].

### 4.6. Role of Oxygen

Prolonged hypoxia’s effect on the development of interstitial pulmonary fibrosis is not specific to COVID-19 but well-documented in the literature [75,76,77] Some studies have suggested a link between hypoxia and the development of pulmonary fibrosis, citing the aberrant interplay between hypoxia, fibroblast formation, and extracellular matrix (ECM) deposition. This been supported by studies showing that hypoxia-inducible factor 1-alpha, (HIF-1-alpha), is implicated in initiation and progression of multiple types of tissue fibroses [75].

By the same token, hyperoxia or prolonged exposure to excessively high amounts of supplemental oxygen has also been documented to lead to PF (DAD histopathology) [78]. This is difficult to mitigate in COVID patients with profound hypoxemia who are susceptible to the more acute effects of tissue hypoxia, but this mechanism is worth considering, especially with regards to growing understanding of what constitutes acceptable oxygen levels in this illness [79].

### 4.7. Other Possibilities

Future investigations into the pathophysiology of post-COVID-19 long term pulmonary disease are urgently needed. These include genetic predisposition and modification of the host microbiota. At the cellular level, TOLLIP (toll-interacting protein) has many roles in the body including regulating inflammation and lung epithelial apoptosis. TOLLIP genetic variants have been implicated in several lung diseases, including IPF, and may provide a novel pathway for understanding chronic sequelae of COVID-19 [80].

Building our understanding of the contributions of alterations to host microbiota in lung disease may also yield valuable insights. Interestingly, acute exacerbations of IPF (AE-IPF) have been associated with alterations in bronchoalveolar lavage (BAL) microbiota burden compared to stable IPF. This shift in respiratory microbiota may also have implications for PCPF [81]. 

## 5. Patterns of Progression of PCPF

One case series documented the need for lung transplantation for post-ARDS fibrosis secondary to COVID-19, but all of these patients had severe ARDS and prolonged MV (>3 weeks). Even after multiple negative virology tests, these patients showed irreversible decline in lung function despite having maximal support with MV and ECMO [82].

PCPF’s course could also be similar to other well-documented forms of post-viral PF such as those occurring after SARS/MERS/H1N1 infection. In SARS patients, post-viral parenchymal damage and patient functional decline mostly recovered within two years of disease onset. CT studies of SARS-CoV-1 showed radiologic features suggestive of fibrosis in more than half of patients after an average of 37 days [83] but interstitial abnormalities in only 5% of patients after 15 years’ follow-up [84]. It is unclear if the change in findings over time represents selection or survival bias as opposed to resolution of fibrotic imaging findings in some patients. Similarly, post-ARDS fibrotic changes have not been seen to progress when the etiology of ARDS is due to viral respiratory infections [13,14]. Even if not progressive, non-reversible fibrotic changes could significantly impact patients’ quality of life, and lead to other disease-related morbidity and mortality [34]. However, there is still much uncertainty about how PCPF may progress; as more data become available, we hope to gain more knowledge on this. 

The fact that there are currently no reliable data on the frequency and severity of PCPF [4,13] may be related to difficulty diagnosing PCPF in current pandemic circumstances. One observational cohort study used follow-up CT scans and diagnosed PF based on extensive radiologic evidence; of 81 survivors of severe COVID-19 pneumonia who had been hospitalized, more than half had radiologic evidence of PF at follow-up [23]. Radiologic features of fibrosis have also been seen in 44% of patients discharged after COVID-19 treatment [85]. Another study showed that there is a high rate of lung function abnormalities on pulmonary function testing suggesting PF on discharge; in this study 47% of patients had impaired gas transfer and 25% had reduced total lung capacity [56]. Finally, in autopsies of thirty patients who died from COVID-19, histopathological progression of DAD to the fibrosing pattern was seen in 43% of samples [25].

## 6. Risk Factors for PCPF

In recent studies, there have been several factors associated with evidence of progression to PCPF in COVID-19 patients. These include older age [23,85,86], profound dyspnea and/or higher respiratory rate [23,85], comorbid hypertension (HTN) [23,85,86], and admission to the intensive care unit [23,85]. Laboratory risk factors for fibrotic features were higher C-reactive protein (CRP) and lower lymphocytes [23,85,86]. In one study of COVID-19 survivors, PF was diagnosed based on extensive fibrotic changes on follow-up CT. This cohort was more commonly male, and their lab differences included leukocytosis, neutrophilia, eosinopenia, and elevated D-dimer. Their clinical course was associated with long prehospital length of fever and in-hospital respiratory failure necessitating supplemental oxygen and/or non-invasive mechanical ventilation (NIMV) [23]. 

A cohort study also showed that lower baseline levels of interferon-gamma (IFN-y) and monocyte chemoattractant protein 3 (MCP-3) at hospital admission for COVID-19 were associated with higher volume of lung fibrosis at discharge. Additionally, there was an autopsy histopathology study done; samples taken were classified as either predominantly fibrosing DAD, acute DAD, or organizing DAD. The fibrosing DAD samples were from patients that had been more than a decade younger, had longer ventilation, and longer hospitalizations [25]. More associations are shown in Table 1.

These findings correlate with risk factors for PF from prior viral pandemics; a follow-up study of MERS survivors showed that the ones who had radiographic evidence of pulmonary fibrosis were older and had longer ICU admissions [87]. Risk factors for post-SARS fibrosis were also older age and likelihood of having been in the ICU [83]. 

## 7. Strategies for Mitigation of Risk

Due to the challenges and lack of effective therapies for treating PF, it is imperative to focus on strategies that aim to reduce the risk of developing PCPF. Such strategies should be directed toward minimizing the factors implicated in perpetuating the cycle leading to persistent lung injury, prolonged inflammatory response, and fibroproliferation [26]. At present, the RNA polymerase inhibitor, remdesivir, is an antiviral agent currently approved for clinical use in treatment of SARS-CoV-2. Initial data have shown some promise in terms of symptom-improvement and resolution of disease in select populations, but is thought to be of greatest benefit to patients early-on in their clinical course and in those with mild-moderate disease [88,89]. However, since it is not known if early viral clearance is protective, the role of remdesivir is not certain.

The exaggerated inflammatory response that occurs in severe COVID-19 infection has also been a target for various medications and therapies, and the use of immunosuppressive agents is presently recommended as part of the standard treatment for COVID-19 infection. The RECOVERY trial has shown moderate-dose dexamethasone given for 10-days decreases the need for and the number of days on MV, reducing the risk of both immune-mediated and iatrogenic lung injury [90]. In addition to therapies targeted specifically at viral and immune-mediated mechanisms, equal attention should be given to mitigating the risk of other modifiable risk factors that are known to increase the risk of pulmonary fibrosis. Ventilator-induced lung injury can be reduced with adherence to lung-protective ventilation strategies, already proven to significantly reduce mortality in patients with ARDS [37].

## 8. Conclusions

A significant number of patients will be at risk for long-term complications following severe COVID-19 pulmonary disease, due to the high prevalence of respiratory failure and the need for MV. The understanding of long-term pulmonary disease in COVID-19 survivors is limited at present time, but increasingly emerging as top priority for the medical community. 

PCPF may be a component of a broader syndrome called post-acute COVID-19 syndrome (PACS). PACS, which has been referred to by other names including “Long Covid”, is a syndrome where patients experience persistent symptoms and/or long-term complications beyond four weeks from the onset of their initial symptoms [74]. These sequelae after disease may include PCPF. Recent studies have shown that persisting symptoms commonly include dyspnea, fatigue or muscle weakness, sleep difficulties, and chest pain, among others [54,74,91]. Evidence suggests that patients who were sicker during their initial hospitalization for COVID-19, especially those requiring high-flow nasal cannula and non-invasive or invasive mechanical ventilation, are more at risk for long term pulmonary sequelae. These may include impaired pulmonary diffusion capacities and abnormalities on imaging suggestive of PF. PACS is an emerging and serious health concern that needs to be addressed [54,74,91].

Different acute phenotypes of COVID have been identified with different clinical courses and outcomes [92]. Similarly, in long term disease, variants among these so-called “long hauler” phenotypes may exist. There may be different post-infectious states among COVID-19 ARDS survivors. Although many will continue to have respiratory symptoms for several months following acute infection, particularly those with underlying asthma, some may recover entirely. Other subpopulations of patients appear to deteriorate three–four weeks after initially getting infected and having a transient recovery. There are also some patients that have very mild courses initially (not requiring medical attention or hospital admission), but weeks later have a resurgence of infectious symptoms and develop ARDS. Because of the early stages of knowledge at this time, a comprehensive review of so called “long hauler” COVID-19 survivors is beyond the scope of this review.

Patients that experience long term cardiopulmonary and neurologic complications following their acute illness may place enormous demands on a healthcare system that is already struggling with limitations in providers and resources. Survivors of COVID-19 who develop persistent pulmonary disease will require long term specialty care; therefore, all clinicians have a vested interest in understanding post-COVID-19 pulmonary fibrosis. It is critical we begin proactively collecting and analyzing objective pulmonary data from COVID-19 survivors in controlled studies in order to identify potentially modifiable clinical risk factors or employ risk mitigation strategies to help protect patients from progression to PCPF. 

## Figures and Tables

**Table 1:** Associations with post-Covid PF. Abbreviations: L/O = length of, LOS = length of stay, f/u = follow-up, RR = respiratory rate, Sx = symptoms, CMs = comorbidities, pts = patients. * = compared to those with acute DAD, ** = compared to all other patterns of DAD. All associations are *p* < 0.05.

Study	N=	Criteria for Defining PF	Age	CMs	Medications	Ventilation	Labs
Yu, et al., 2020	32	Based on f/u CT; Pts put into 1 of 2 groups. Fibrosis group had evidence of fibrosis.	Older (Median 54 y.o, vs. 37)	HTN	Longer L/O steroid therapy, longer L/O antiviral therapy	–	Higher CRP, higher IL-6, lower lymphocytes
Hu, et al., 2020	76	Based on CT at discharge; Pts put into 1 of 2 groups. Fibrosis group showed presence of PF.	Older (Median 58 y.o, vs. 39)	HTN	–	–	Higher CRP, lower IFN-y, lower lymphocytes
Huang, et al., 2020	81	Based on f/u CT; Pts put into 1 of 2 groups: PF group showed fibrotic changes.	Older (Median 63 y.o, vs. 51)	HTN	More likely to have needed steroids	Higher rate of ventilation	Higher CRP, higher D-Dimer, lower lymphocytes
Li, Y. et al., 2020	30	Based on histopathology; Samples put into fibrosing DAD, acute, or organizing groups.	Younger * (Median 64 y.o, vs. 77)	–	–	Longer ventilation **	–
